# Coproducing Science to Inform Working Lands: The Next Frontier in Nature Conservation

**DOI:** 10.1093/biosci/biz144

**Published:** 2019-12-18

**Authors:** David E Naugle, Brady W Allred, Matthew O Jones, Dirac Twidwell, Jeremy D Maestas

**Affiliations:** 1 Wildlife biology, the University of Montana (UM), Missoula, and is the national science advisor for Working Lands for Wildlife, part of the USDA’s Natural Resources Conservation Service. Also at UM; 2 Rangeland Analysis Platform in the W. A. Franke College of Forestry and Conservation; 3 Ecologist in UM’s Numerical Terradynamic Simulation Group; 4 Rangeland resiliency, Center for Resilience in Agricultural Working Lands and the Department of Agronomy and Horticulture, the University of Nebraska, Lincoln; 5 Sagebrush ecosystem specialist with the Natural Resources Conservation Service, Portland, Oregon

**Keywords:** Conservation outcomes, coproduction, Farm Bill, partnerships, private lands

## Abstract

Conservationists are increasingly convinced that coproduction of science enhances its utility in policy, decision-making, and practice. Concomitant is a renewed reliance on privately owned working lands to sustain nature and people. We propose a coupling of these emerging trends as a better recipe for conservation. To illustrate this, we present five elements of coproduction, contrast how they differ from traditional approaches, and describe the role of scientists in successful partnerships. Readers will find coproduction more demanding than the loading dock approach to science delivery but will also find greater rewards, relevance, and impact. Because coproduction is novel and examples of it are rare, we draw on our roles as scientists within the US Department of Agriculture–led Sage Grouse Initiative, North America's largest effort to conserve the sagebrush ecosystem. As coproduction and working lands evolve, traditional approaches will be replaced in order to more holistically meet the needs of nature and people.

Two emerging trends in natural resource conservation are the coproduction of science to increase its utility in policy, decision-making, and practice (Lemos et al. 2018) and a renewed reliance on working lands (i.e., privately owned in agricultural production) to sustain nature and people (figure 1; Kremen and Merenlender 2018). The coproduction of knowledge is a way of making science more actionable by engaging stakeholders to share in its design and implementation, with a focus on achieving better outcomes for society (Lemos et al. 2018). Popular with sociologists since the 1970 s as a way to set inclusive public policy (Bovaird 2007), coproduction is now mainstream in the sustainability sciences (Willyard et al. 2018), including climate research (Bremer and Meisch 2017). Notable is the recent popularity of coproduction in health care with patient and public involvement leading to better outcomes (Hickey et al. 2018). Although it is more self-evident why coproduction works, scholars are still figuring out how to best implement it broadly by uncovering commonalities in practice and operationalizing it up and down all levels of governance (Garmestani et al. 2019).

**Figure 1.**
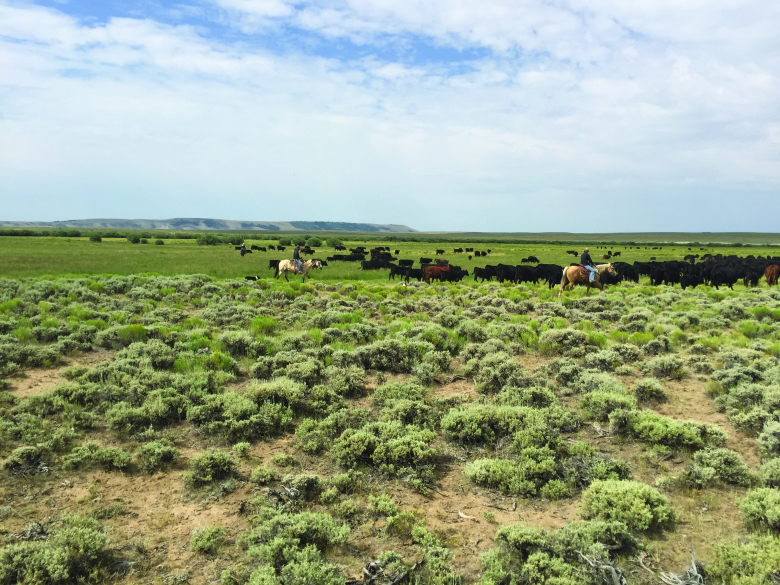
Productive working lands that support wildlife and people in the western sagebrush ecosystem.  Photograph: Mandi Hirsch.

Commensurate with the rise of coproduction (Gerber and Raik 2018) is a societal awakening to the value of working lands as important reservoirs of primary productivity (Robinson et al. 2019) and carbon sequestration (Griscom et al. 2017), conservators of biodiversity and ecosystem services (Brockington et al. 2018), and the connective tissue that maintains resiliency of nature reserves (Mahoney et al. 2015). Moreover, working lands bolster the ecosystem values of existing natural areas (Drescher and Brenner 2018) and help to conserve conservation-reliant species for which persistent threats cannot be eliminated but only actively managed (Goble et al. 2012). If widely adopted for working lands, coproduction could provide participants with the knowledge necessary to better sustain rural livelihoods and nature's resources on privately managed rangelands, forests, and cultivated lands that collectively occupy 80% of global terrestrial area.

Coproduction is catching on in academic institutions experimenting with new institutional approaches for achieving coproduced outcomes between university researchers and the general populous. Although it is too early for widespread adoption, emerging themes include universities serving as boundary organizations to multiple nongovernmental organizations seeking to ramp up their science capacity and colleges hosting cosponsored scientists on campus, such as in The Nature Conservancy's Professors of Practice program (Gerber and Raik 2018). Although an area of potential academic growth, very few universities are training the next generation in working lands conservation or integrating concepts of coproduction into course offerings. This academic void in most curricula today is reminiscent of the period immediately preceding the emergence of human dimensions studies around 20 years ago (Manfredo et al. 2004) as conservationists realized that natural resource management is mostly people management.

Scientists, natural resource managers, and funders (Beier et al. 2017) are increasingly convinced that collaborating to coproduce knowledge brings durability to policies and actions. So much so that some funding opportunities mandate meaningful scientist–stakeholder engagement, and institutions have begun evaluating researchers by their level of social impact (Chubb and Reed 2018). No doubt, the future for coproduction lies in creating new structures that foster deeply integrated knowledge across disciplines and readily accessible by nonacademic stakeholders (Irwin et al. 2018).

Despite these trends, coproduction of science to inform conservation of working lands remains rare. Calls abound (Lemos et al. 2018) for scientists versed in actionable science to engage in helping assess outcomes of land management and to improve society's return on investment in conservation. To do so, science needs to be done with the intent to deliver conservation actions, and delivery should be done with the intent to measure outcomes relevant to society at large (Burger et al. 2019). Coproduction recognizes that much science has been done in the name of society but has not been used by society; the traditional science-delivery model is not as relevant in today's world, and the tendency for science to be paid for and produced, dutifully published, and then left on the shelf for someone else to find and use is no longer a suitable communication strategy (Nature editorial 2018). Instead, the coproduction model of linking science with decision-making is more demanding than the loading dock approach to science delivery (Cash et al. 2006), but the rewards for scientists are many, including increased relevance and impact. Furthermore, the advancement of this pursuit would benefit from specific examples of how coproduction has been applied to working lands so that interested members of the scientific community can learn from those pursuits and engage as key partners of professional conservation teams.

In this Professional Biologist article, we illustrate the marriage of coproduction with working lands conservation (figure 2), present five elements of a successful coproduced approach (table 1), and suggest to professional biologists their potential roles in each. Readers will find that our motivations are purely normative; we deliberately chose to coproduce knowledge inclusive of diverse perspectives (Lemos et al. 2018). Where they are obvious, we identify instances in which our efforts are shaping the broader societal narrative in working lands conservation but aptly leave more descriptive pursuits to the true scholars of coproduction (e.g., Wyborn et al. 2019). We use the terms scientist and biologist interchangeably in the present article to describe individuals who the group relies on for technical expertise in the coproduction of science. We do so from our perspective as science advisors to the US Department of Agriculture's (USDA) Working Lands for Wildlife effort. Specifically, we use a case study from the USDA Sage Grouse Initiative (SGI), a decade-long effort to conserve North America's vast sagebrush (Artemisia spp.) ecosystem.

**Figure 2.**
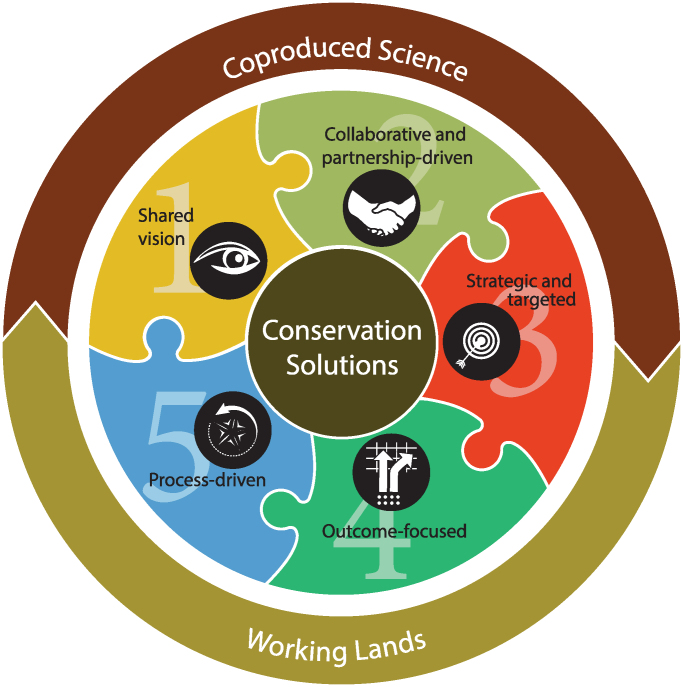
The marriage of coproduction and working lands conservation.

**Table 1.** Five elements of a successful coproduced, working lands conservation approach compared with the more traditional scientific process.

Traditional Approach	Coproduction Approach
Reactive: Scientists engage late in the process, and often in response to litigation or crisis	Shared vision: Scientists engage early in building a shared vision of desired outcomes for ecosystems and people
Stove piped and agency specific: Science largely confined to within-agency directives	Collaborative and partnership driven: External scientists invited to help shape conservation program delivery
Planning averse and opportunistic: Scientists operate outside of conservation program delivery, isolating themselves from real world applications	Strategic and targeted: Scientists inform strategic plans and help target resources to maximize return on investment
Output focused: Managers simply track and report on conservation actions or dollars spent with no role for science	Outcome focused: Managers work closely with scientists to quantify outcomes of conservation actions
Product driven: Science ends with peer-reviewed publication; scientists leave to pursue the next funded study	Process driven: Science continues within the evolving partnership by improving program delivery, identifying emerging opportunities and implementing conservation solutions

## Working lands for wildlife

Greater sage grouse (Centrocercus urophasianus) populations have declined 0.83% annually since 1965 (Western Association of Fish and Wildlife Agencies 2015) largely because of habitat loss and fragmentation (Schroeder et al. 2004). Its primary impacts include energy and infrastructure development (Allred et al. 2015), wildfire and exotic annual grasses (D’Antonio and Vitousek 1992), cultivation (Lark et al. 2015), woodland expansion (Romme et al. 2009), and residential development (Hansen et al. 2002). In 2010, the USDA Natural Resources Conservation Service launched the SGI to help agricultural producers voluntarily reduce threats facing the sage grouse on working rangelands across the western United States. In 2012, the SGI served as the flagship for the establishment of Working Lands for Wildlife, an effort to conserve other at-risk ecosystems and associated species. The agency employs the $60 billion conservation title of the federal Farm Bill legislation (title II of the Agriculture Improvement Act of 2018, FY2019–FY2023) to help landowners voluntarily implement conservation practices on private farms, ranches, and forestlands.

Quantifying the outcomes of the resulting conservation for species and ecosystems and the iterative use of emerging science to improve delivery are integral components of Working Lands for Wildlife. Over the last decade, the SGI has matured into a primary catalyst for science-driven sagebrush conservation by using Farm Bill resources to restore or enhance more than 28,700 square kilometers (km^2^) of sage grouse habitat on more than 1800 ranches while supporting sustainable agricultural productivity on these working lands. The most recent listing decision served as a litmus test for whether voluntary conservation would be viewed on par with regulatory mechanisms in a federal listing determination. Indeed, in 2015, sage grouse were determined to not warrant protection under the Endangered Species Act, with the SGI Outcomes in Conservation report describing working lands conservation efforts (Natural Resources Conservation Service 2015) cited 43 times in the Federal Register listing decision (USDOI 2015). With Working Lands for Wildlife now codified nationally in the new Farm Bill, we write this article to stimulate deliberation on ways to continually improve the melding of coproduced science and perhaps take deliberate steps to institutionalize it within working lands conservation (Wyborn et al. 2019).

### Element 1: Shared vision

Scientists involved with coproduction can engage conservation practitioners early by helping build a shared vision of the desired outcomes for ecosystems and people (table 1, figure 2). Scientists contemplating coproduction should consider whether their ethos generally aligns with the partnership's shared vision (Oliver et al. 2019). An agreed on vision in working lands is typically focused on implementing conservation that improves the landowner's own sustainability by reducing persistent ecosystem-level threats. This proactive and voluntary element is often well received when compared with reactive and often litigious actions that pervade imperiled species management (e.g., US federal Endangered Species Act regulations). The SGI’s shared vision of wildlife conservation through sustainable ranching includes ranchers as part of the solution for implementing practices that reduce persistent, nonregulatory threats (USFWS 2013). Getting the shared vision right from the start rallies and sustains partnerships, whereas getting it wrong can jettison the best of intentions. People constantly question how the SGI is able to sustain landowner enrollment and how we get landowners to participate. This is where a shared vision is crucial to the sustainability of coproduction (i.e., good for the bird, good for the herd). No one is forced to enroll; instead, the SGI’s vision is congruent with ranchers’ values and leverages the Farm Bill's 80-year history of voluntary conservation to put that shared vision into practice.

Membership by conservation professionals does not compromise autonomy as independent, third party assessors that advise objectively, especially when coproduced science does not yield anticipated outcomes. Such was the case when pastures rested from domestic grazing did not benefit sage grouse populations as originally hypothesized (Smith et al. 2018a), and in response, funding agencies adjusted the delivery of conservation practices to de-emphasize financial incentives being paid for extended rest within rotational grazing systems. Findings spawned additional inquiry challenging the long-held belief that grazing restrictions inevitably benefit sage grouse populations. A follow-up study revealed that commonly used methodologies were inherently biased, misrepresenting the relationships between habitat structure and sage grouse nest success (Smith et al. 2018b). These results, along with the ranchers’ traditional ecological knowledge (e.g., Berkes et al. 2000) of this system initiated, a third line of questioning to understand the economic implications of the unintended habitat loss on private land resulting from grazing restrictions on publicly adjacent rangelands (Runge et al. 2019). Collectively, this string of coproduced science, brought together by a shared vision, is raising the collective appreciation of the more complex interrelationships between wildlife habitat and ranching enterprises in this public–private checkerboard of land ownership of the western United States.

### Element 2: Collaborative and partnership driven

Many collaborative partnerships are typically landowner led (e.g., the Blackfoot Challenge, the Malpai Borderlands Group, the Tallgrass Legacy Alliance, the Sandhills Task Force), and stakeholders, including scientists, are vetted for their expertise and collaborative skill sets. Initial trust and credibility is deeply rooted within local landowner leaders, which is critical for the longevity of the resulting conservation (Duvall et al. 2017). Like-minded, landowner-led groups are coalescing into umbrella organizations to extend their shared vision of working lands conservation into additional watersheds (e.g., Western Landowners Alliance, Partners for Conservation). Involved scientists can proactively help shape the delivery of these collaborations by putting state-of-the-art scientific technologies into the hands of private and public partners. Recurring themes include providing both the spatial context for efficient allocation of resources (Bottrill et al. 2008) and the rigorous use of the best available science in selection and implementation of conservation practices. For the SGI, the species’ occupied range is daunting (271,000 km^2^), so scientists conducted spatial analyses showing that 75% of the birds are clumped within 27% of their 11-state breeding range (Doherty et al. 2010). Within these newly identified sage grouse strongholds, the partnership strategically placed additional human capacity for delivery of programs, a critical but often missing piece in conservation (Bennett et al. 2018). Within the first 5 years, these shared positions, which were funded in part by the Farm Bill and matched by more than 40 contributing partners, have yielded more than 11,100 additional field visits with landowners that ultimately doubled the acreage of completed conservation (Natural Resources Conservation Service 2015). SGI scientists also shared their knowledge of the best available science with regulators in months-long meetings that resulted in approved practices to restore and enhance bird habitat—again, a necessary but atypical role for most academics. After the launch of the SGI, these efforts morphed into the pioneering concept of regulatory predictability meaning that, under the authorities granted to the US Fish and Wildlife Service by Endangered Species Act legislation, enrollees are exempt from incidental take requirements if the species is listed and affected inadvertently by the implementation of approved conservation practices (USDA 2014). The predictability with federal regulatory agencies has the potential to rebuild trust in government, which, in turn, buoys landowner participation by programmatically granting certainty that the enrollees will not be asked to do more if the species is later listed.

### Elements 3 and 4: Strategic, targeted, and outcome focused

The dual role of coproduced science is to develop spatial targeting tools to pinpoint where to invest in conservation (element 3) and to evaluate whether investments yield the desired outcomes (element 4). Scientists versed in coproduction know well the painstaking preplanning and delayed gratification that accompanies habitat manipulations to quantify outcomes that do not measure themselves. SGI partners recognized early that limited resources necessitate a strategic, landscape-scale approach that replaces random acts of conservation kindness to increase the odds of achieving desired outcomes. Rather than adopt archaic reporting requirements (e.g., acres enrolled, miles protected, or dollars allocated), the SGI instead employed targeting tools and outcome-based assessments that informed and adapted on-the-ground conservation. For example, managing expanding woodlands (i.e., juniper, Juniperus spp.) is an SGI practice that has helped landowners restore more than 3150 km^2^ of sagebrush rangelands with beneficial outcomes for wildlife, vegetation, water, and associated ecosystem services (Miller et al. 2017). To target this practice, the SGI coproduced the first high-resolution mapping of tall woody plant cover across sagebrush habitats (Falkowski et al. 2017) and made this tool freely available via the SGI interactive web application for partners to identify areas of early tree invasion and visualize potential areas in need of treatment. Outcomes from the SGI’s decade-long evaluation in Oregon showed that restored habitats were rapidly recolonized by sage grouse (Severson et al. 2017a) with higher survival rates inside than outside of treatments (Severson et al. 2017b), resulting in a 12% increase in population growth rate (Olsen 2019). Songbird abundances also doubled with management (Holmes et al. 2017), providing umbrella benefits to other imperiled wildlife across the Great Basin (Donnelly et al. 2017).

### Element 5: Process driven

Embracing the entirety of coproduction enables science to inform delivery, resulting in enhanced working lands conservation. We impress on readers that these five elements are more likely to yield desired outcomes when used in concert, that no single element is more important than another, and that effectiveness is lost when decoupled (figure 2). Unlike traditional scientific pursuits, coproduction continues after scientific publication. Explicit in the process is science transfer to field and lay audiences, which catapults conservation effectiveness. The SGI is dedicated to transferring actionable science to field practitioners, as is evidenced by its development of web applications (Rangeland Analysis Platform, https://rangelands.app), field trainings, workshops, and webinars. Communicating conservation outcomes is critical to continued success but entails actions, skill sets, and time commitments that many scientists occupying more traditional roles may be unaccustomed to. When coproduced outcomes are available, demystifying science through communications in layperson's terms becomes a luxury rather than a burden and a mechanism for sustained investment because the partners are able to articulate a return on investment to the stakeholders. Coproduction itself is a process-driven pursuit by which scientists can engage the broader citizenship and escape disciplinary tendencies to focus on a single idealized resource target known to constrain long-term effectiveness of conservation practice (Twidwell et al. 2013).

On the other hand, coproduction has its own opportunity costs and caveats that, if not guarded against, can reduce coproduction to an end in and of itself (Lemos et al. 2018). Although we recognize the critical nature of advancing scientific theory, the SGI intentionally maintains limited expertise in the basic sciences to prevent mission drift and reduces costs by instead contracting with outside specialists, thereby maintaining the quality and rigor of the resulting targeting tools and outcome-based assessments. Such collaborations also reduce the potential for exclusivity that could otherwise result from the persistent and selective interaction of the same groups of scientists and stakeholders. Evidentially, the SGI has contributed both locally and globally to the collective knowledge base through peer review with 11 different university scientists, 11 more state and federal agency biologists, and 7 industry and nongovernmental conservation organizations. We readily admit that the SGI does not always coproduce science but instead sometimes collates and coassesses with stakeholders the utility of existing knowledge, coproducing new knowledge to fill in the gaps and to test its efficacy in novel geographies (Sutherland et al. 2017).

## Conclusions

We hope that fellow biologists add to, reshape, and mold the elements identified in the present article to increase their transferability in furthering working lands conservation. At the SGI, we often jest that science chases implementation because of our partners’ appetites for access to coproduced science, online tools, and additional outcome-based evaluations. The Farm Bill is one of the largest funding mechanisms for conservation in the world, but, with its relative obscurity to many in conservation, much work remains to raise awareness of its importance to the next generation. In the present article, we identify an additional role for future scientists to contribute to scientific use in society, adding to the already existing good works of colleagues in the policy-based and basic sciences. Moving forward, we encourage university and agency administrators to identify innovative pathways for academic faculty and agency researchers to participate in working lands partnerships as essential and appreciated components of professional working teams. As coproduction gains traction, real-life case studies, such as the story of the SGI, are fertile ground for learning, in real-time, how to most effectively seed power sharing up and down all levels of governance. Only then will coproduction move beyond the creation of science and into broader contexts in which that knowledge is used with a clear normative objective to support societal change.

## Author Biographical

David E. Naugle (david.naugle@umontana.edu) is a professor in wildlife biology at the University of Montana (UM), in Missoula, and is the national science advisor for Working Lands for Wildlife, part of the USDA’s Natural Resources Conservation Service. Also at UM, Brady Allred is a professor of rangeland ecology and architect of the Rangeland Analysis Platform in the W. A. Franke College of Forestry and Conservation. Matthew O. Jones is an applied ecologist in UM’s Numerical Terradynamic Simulation Group. Dirac Twidwell is a professor of rangeland resiliency in the Center for Resilience in Agricultural Working Lands and the Department of Agronomy and Horticulture at the University of Nebraska, in Lincoln. Jeremy D. Maestas is a sagebrush ecosystem specialist with the Natural Resources Conservation Service, in Portland, Oregon.
